# Petal-like Patterning of Polylactide/Poly (Butylene Succinate) Thin Films Induced by Phase Separation

**DOI:** 10.3390/polym15224463

**Published:** 2023-11-20

**Authors:** Lili Wang, Yujie Wang, Chudi Mou, Wanjie Wang, Chengshen Zhu, Suqin He, Hao Liu, Wentao Liu

**Affiliations:** 1 School of Materials Science and Engineering, Zhengzhou University, Zhengzhou 450001, China; iceblessom@163.com (L.W.); yjwang@haue.edu.cn (Y.W.); n110505@163.com (C.M.); wwj@zzu.edu.cn (W.W.); zhucs@zzu.edu.cn (C.Z.); hesq@zzu.edu.cn (S.H.); 2 School of Chemical and Printing-Dyeing Engineering, Henan University of Engineering, Zhengzhou 451191, China

**Keywords:** polylactide, poly (butylene succinate), phase separation, microcrystals, nanomechanical

## Abstract

Biodegradable plastics are attracting attention as a solution to the problems caused by plastic waste. Among biodegradable plastics, polylactide (PLA) and poly (butylene succinate) (PBS) are particularly noteworthy because of their excellent biodegradability. However, the drawbacks of their mechanical properties prompts the need to compound them to achieve the desired strength. The characteristics of the interface of the composite material determine the realization of its final performance. The study of the interface and microstructure of composites is essential for the development of products from degradable polymers. The morphology evolution and microcrystal structure of spin-casted fully biodegradable (PLA/PBS) blend films were investigated using atomic force microscopy (AFM)-based nanomechanical mapping. Results show that intact blend films present an obvious phase separation, where the PBS phase is uniformly dispersed in the PLA phase in the form of pores. Furthermore, the size and number of the PBS phase have a power exponential relationship and linear relationship with PBS loading, respectively. Intriguingly, after annealing at 80 °C for 30 min, the PLA phase formed an orderly petal-like microcrystalline structure centered on the PBS phase. Moreover, the microcrystalline morphology changed from a “daisy type” to a “sunflower type” with the increased size of the PBS phase. Since the size of the PBS phase is controllable, a new method for preparing microscopic patterns using fully biodegradable polymers is proposed.

## 1. Introduction

For decades, considerable attention has been paid to polylactide (PLA) [1,2,3] due to its renewability, biodegradability, and relatively lower cost, which make PLA an ideal substitute for petroleum-based polymers. Nevertheless, drawbacks such as relatively low impact strength, heat distortion temperature, crystallization rate, etc., limit its widespread application. Accordingly, huge amounts of research focuses on PLA-based composites [4,5,6,7,8,9]. In view of the fact that PLA-based films have abilities such as a controllable morphology, porosity ratio, and adherence to flexible substrates, PLA-based thin films have potential applications in numerous areas like biomembranes [10,11], protective coatings [12], sensors [13,14], and active layers in devices [15].

During the fabrication of polymer thin films, dewetting [16,17] and phase separation [18] often take place simultaneously and constantly. Dewetting occurs on a non-wetting solid surface as a result of the polymer chains possessing enough mobility, which leads to an unstable or metastable thin polymer film [19]. Phase separation forms when the solvent evaporates from its solution and generates various morphologies, which can be controlled by many factors, such as the solvent, the substrate, film thickness, etc. [20,21]. In a recent work of Bin Zhang and coworkers [22], flow-induced dendritic β-form isotactic polypropylene (iPP) lamellae in thin films were obtained via isothermal crystallization after scratching supercooled iPP melt with a sharp scalpel, during which a very high nucleation density of edge-on α-iPP crystals oriented perpendicular to the flow direction were generated. In another study by Zhang et al. [17], highly stretched filaments of ultrahigh molecular weight polyethylene were obtained via the dewetting of a spin-coated film of hot p-xylene solution on a silicon substrate, with partial decoration of edge-on lamellar crystals due to the shear forces during dewetting, resulting in so-called shish-kebab structures. Even after annealing and subsequent quenching to room temperature, these filaments still present shish-kebab structures as a result of the memory of the dewetting-induced stretching kept by polymer chains, which were confined in nanoscopic filaments and underwent slower conformational changes and rearrangement processes than those observed in an equilibrated melt. Guangwu Guan’s work [23] shows that a patterned poly(l-lactide) structure with two fixed lamella and chain orientations can be observed when the poly(l-lactide) layer is deposited on a uniaxially-oriented isotactic iPP substrate. The crystallization behavior of poly(l-lactide) is demonstrated by the major lamellar set, which is oriented with molecular chains perpendicular to the chain direction of the iPP, while the minor lamellar set is inclined at about 64° to both the iPP chain axis direction and the lamellae of the major set. The reason for the orientation of the main set is that poly(l-lactide) chains are oriented parallel to the ditches of the iPP substrate caused by alternatively arranged crystalline and amorphous regions, while those of the minor set are a homoepitaxy of PLLA with parallelism of the helical paths. It has been confirmed that the shear force occurred during both dewetting processes and the flow field should sharply reduce the free energy barrier of crystal nucleation and induce the formation of an oriented pattern or crystal in polymer films [24,25,26,27], which make it predictable that the crystalline polymer films display similar behavior because of the shear force driven by phase separation [28].

PBS has been industrially produced for many years as a valid, sustainable, bio-based, and biodegradable plastic [29]. Compared with PLA, it possesses excellent processability [30] and good thermal stability [31]. Typically, PBS is used as a ductility enhancer for PLA to improve its toughness [32]. Several works reported that PLA/PBS blends could effectively enhance the toughness, such as the elongation at breakage and the impact strength, with a relatively low content of PBS [33,34,35].

The main objectives of the present study are to discuss the morphology evolution of the PLA/PBS thin films with different annealing temperatures and times, which were obtained by solution spin casting with different PBS contents, and characterized by AFM at room temperature.

## 2. Materials and Methods

### 2.1. Materials

The PLA (4032D) used in this study was purchased from Natureworks (Minneapolis, MN, USA) with a *M_w_* of 210 kDa and density of 1.24 g/cm^3^. The PBS (3001MD) was purchased from Bionolle (Tokyo, Japan) with a *M_w_* of 120 kDa and density of 1.23 g/cm^3^. The glass transition temperature (*T_g_*) and melt temperature (*T_m_*) of PLA were 63.5 °C and 165 °C, while the *T_m_* of PBS was 92 °C, determined with Differential Scanning Calorimeter (DSC) (DSC204, NETZSCH, Selb, Germany), respectively. The tests were carried out under nitrogen protection using a single heating procedure that started at room temperature and heated up to 210 °C at a rate of 10 °C/min.

Anhydrous ethanol, acetone, and trichloromethane were purchased from Tianjin Fengchuan Chemical Reagent Technology Co. (Tianjin, China), both of which were Analytical Reagents. Monocrystalline silicon wafer with (100) crystal form was purchased from Zhejiang Lijing Silicon Materials Co. (Quzhou, China).

### 2.2. Fabrication of PLA/PBS Blend

The PLA/PBS blend was prepared at different composition ratios (PLA/PBS: 100/0, 90/10, 85/15, 80/20, 75/25, 70/30 by weight) using a CTR-100 torque rheometer (Shanghai Changkai Electromechanical Technology Co., Shanghai, China). Raw materials should be dried in vacuum oven at 60 °C for 24 h. All of the three heating zones of the torque rheometer were set to 190 °C for preheating for about one hour. Melt blending began after the instrument temperature was stabilized.

### 2.3. Fabrication of PLA/PBS Blend Thin Films

Preparation of spin-coating substrate. Monocrystalline silicon wafer was cut into squares of 1 × 1 cm^2^ as spin-coating substrates. The substrates were successively ultrasonically cleaned in anhydrous ethanol and acetone for 20 min and then dried in vacuum oven.

Preparation of solution. Samples were obtained as in step 2.2 using an analytical balance and were placed into 5 mL clean volumetric flasks. Then, we added trichloromethane to the scale mark of the volumetric flasks. We let it stand until the samples were completely dissolved, followed by ultrasonic treatment for 10 min to ensure the mixture was homogeneous. The concentrations of neat PLA solution were 5 mg/mL, 10 mg/mL, and 20 mg/mL, while that of the blend solution was 20 mg/mL.

Fabrication of PLA/PBS blend thin films. All kinds of films were achieved by spin-coating the solutions onto pre-cleaned silicon wafers using a KW-4A spin-coater (Institute of Microelectronics, Chinese Academy of Sciences, Beijing, China), while the spin speed and time were 3000 rpm and 40 s, respectively. The experiment must be carried out in a fume hood.

### 2.4. Process and Conditions of Crystallization

The samples chosen for crystallization investigation were placed in vacuum ovens for annealing. The temperature of each oven was 40 °C and 80 °C. And the crystallization times of different groups were 30 min and 12 h, respectively. All the samples were cooled to room temperature naturally after annealing. 

### 2.5. Characterization of PLA/PBS Blend Films

#### 2.5.1. X-ray Diffraction (XRD)

The crystalline characteristics of the thin film samples were tested using XRD. The X-ray Diffractometer (X′pertPRO, PANalytical B.V., Almelo, Netherlands) had a copper target at the emitter end with a wavelength of 1.54 Å. All the tests were carried out with scanning angle range of 5–30° and scanning speed of 5 °/min. And before the test began, a blank silicon wafer should be used to press the plasticine into the center of the sample stage to a proper height.

#### 2.5.2. Micromorphology

All the morphology characterizations of samples were performed via atomic force microscopy (AFM) in tapping mode on a Bruker Multi Mode 8 SPM (Billerica, MA, USA) under ambient conditions at room temperature. The probes chosen for tapping mode were Tap300Al (Budget Sensors, Sofia, Bulgaria) probes with a spring constant of 40 N/m and a resonance frequency of 300 kHz. The scanning area was randomly selected in the central part of the film and different scanning sizes were selected in order to obtain the morphology of the film at different scales. In addition, the thickness of the film was determined by making a cross mark in the center part of the film using the tip of a pair of tweezers and by testing the edge of the mark to measure the height difference between the film surface and the silicon substrate.

#### 2.5.3. Nanomechanical Mapping

In order to better understand and determine the two-phase distribution, blend films were tested using nanomechanical characterization. The nanomechanical characterization is based on the force spectra of the AFM, which allows one to obtain the distribution of the isotopic nanomechanical properties of the samples while scanning their microscopic morphology. The nanomechanical properties were tested in FV (Force Volume) mode using a Multi75Al-G (Budget Sensors, Sofia, Bulgaria) probe with a spring constant of 3 N/m at a resonance frequency of 75 kHz. Firstly, a TiO_2_ standard sample in the tapping mode was scanned to obtain the radius of the probe tip. Subsequently, a sapphire standard sample was scanned in FV mode to obtain the deflection sensitivity of the probe cantilever, and the spring coefficient of the probe was measured using the thermal tuning method. Finally, the samples to be tested were replaced on the scanner and scanned for nanomechanical properties at a randomly selected 128 × 128 dot matrix. The TiO_2_ standard sample needed to be scanned again using the same probe after all the tests were completed to ensure that there was no significant change in the radius of the probe before and during the test.

Force Volume (FV) mode of AFM can be used to obtain the probe deflection-piezoelectric scanner displacement curves at different positions of the sample, which can be converted into force–deformation curves using the following equation:

*F = kd* (1)

*δ = Z − Z_0_ − d* (2)

where *F*, *k*, *d*, *δ*, *Z*, and *Z*_0_ in the above equations refer to the force, spring cantilever constant, cantilever deflection, deformation length, piezoelectric scanner displacement, and displacement of the piezoelectric scanner located at the tip of the cantilever beam and at the level of the surface of the undisturbed sample, respectively.

**Figure 1:**
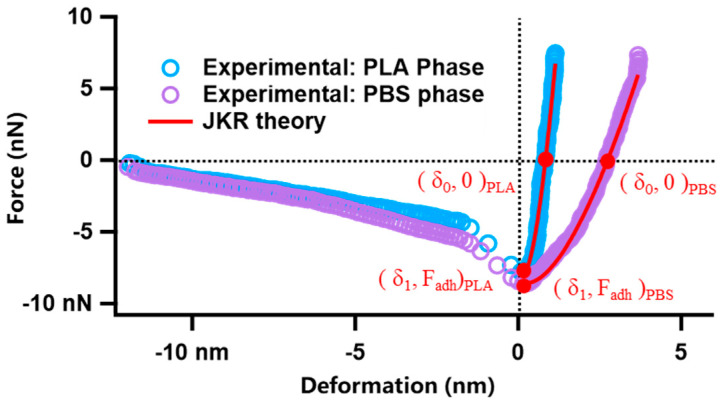
Force−deformation curves of PLA, PBS, and JKR fitting results.

The nanoscopic Young’s moduli of the samples can be calculated from the force–displacement curves obtained by applying the appropriate contact mechanics theory. It has been shown that the Johnson–Kendall–Roberts (JKR) adhesion contact theory is applicable to the vast majority of polymer systems. The JKR contact theory can be explained by the following equation:

(3) $$F=\frac{K}{R}a^{3}-\;3w\pi R\;-\sqrt{6w\pi \mathrm{RF}+{\left(3w\pi R\right)}^{2}}$$

(4) $$\delta =\frac{a^{2}}{3R}+\frac{2F}{3aK}$$

where *a*, *R*, *W*, and *K* are the contact radius, the probe tip radius, the adhesion energy, and the converted Young’s modulus, respectively. Young’s modulus *E* can be calculated from the following equation:

(5) $$E=\frac{3\left(1-\nu^{2}\right)}{4}$$

where *ν* is the Poisson ratio of the material. Since JKR theory has two equations and three unknowns, it is impossible to solve the value of *E* with linear fitting. But the “two-point method” proposed by Sun et al. [36] was applied to solve this problem, i.e., two special points in the JKR curve were taken: the point where the adhesion force is equal to the repulsive force and the point of the maximum adhesion force, as shown in Figure 1. When the JKR curve takes the above points, the JKR equation is transformed into the following equation, where *K* can be represented explicitly as

(6) $$K=\frac{1{.27F}_{1}}{\sqrt{R{\left(\delta_{0}-\delta_{1}\right)}^{3}}}$$

According to Equations (4) and (5), it is possible to obtain the value of *K* derived from the force–deformation curves. The values of Poisson’s ratio of PLA and PBS can be obtained from the literature, from which all of the nanoscopic Young’s moduli of PLA/PBS blend film samples can be calculated [37].

## 3. Results and Discussion

### 3.1. Microstructure of PLA Film

**Figure 2:**
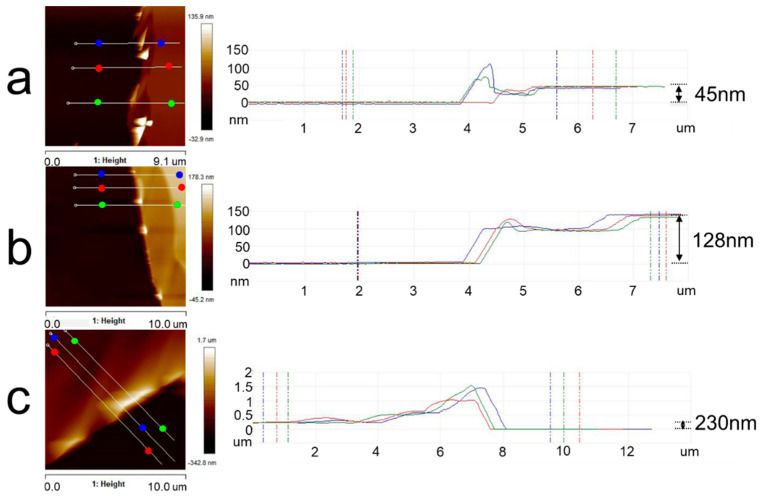
AFM topography images of PLA thin films and an analysis of film thickness with different concentrations of PLA solution: (**a**) 5 mg/mL; (**b**) 10 mg/mL; (**c**) 20 mg/mL. The solvent was chloroform. The right graphs indicated the height between two points of the same color in the corresponding left images.

The thickness of the polymer film is mainly controlled by solution concentration, and it is necessary to determine the appropriate concentration of the copolymer solution before spin-coating. Figure 2 mainly shows the analysis of PLA film thickness with different solution concentrations. The thickness of PLA films could be obtained by calculating the height difference between the film surface and the silicon substrate. Those highlighted areas in the height images of PLA films were stacked or curled film debris scratched by sharp tweezers. When the solution concentrations were 5 mg/mL, 10 mg/mL, and 20 mg/mL, the thicknesses of PLA films were around 45 nm, 128 nm, and 230 nm, respectively. The typical thickness of thin films always exceeds 100 nm, while that of ultrathin films is smaller than 100 nm [38]. Obviously, the concentration of 20 mg/mL could be a better choice.

**Figure 3:**
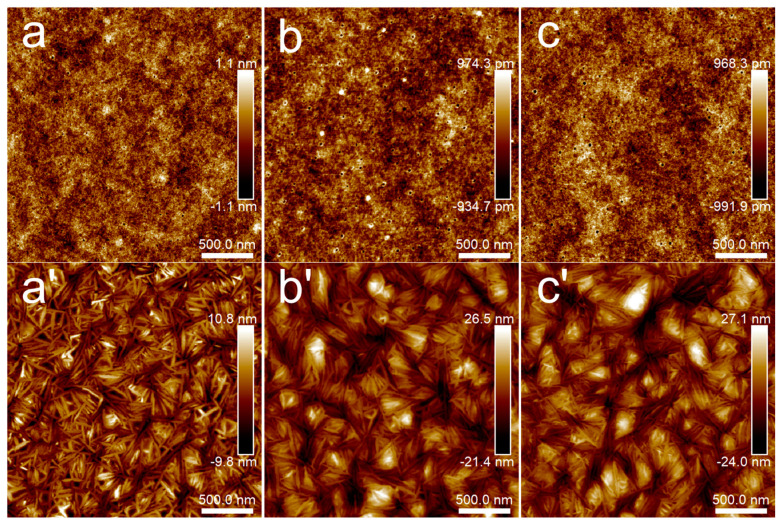
AFM topography images of PLA thin films: (**a**,**a’**) 5 mg/mL; (**b**,**b’**) 10 mg/mL; (**c**,**c’**) 20 mg/mL. Groups (**a**–**c**) were samples without annealing, while the annealing temperature and time of groups (**a’**–**c’**) were 80 °C for 30 min. The scan size was 2.5 × 2.5 μm^2^.

Figure 3 presents the microstructure of PLA films with different film thicknesses at room temperature and after annealing at 80 °C for 30 min. It can be seen from the height images that each PLA film at room temperature had a relatively flat surface, and the height difference was about 2 nm. After annealing at 80 °C for 30 min, the microcrystal structure of PLA appeared and the microcrystal distribution was disordered. When the thickness of the PLA film was about 45 nm, the height difference of the microcrystal structure was about 20 nm. When the film thickness exceeded 100 nm, whether 148 nm or 230 nm, the height difference of the microcrystalline structure increased and was maintained around 50 nm. In other words, the film thickness had some influence on the height difference of PLA microcrystals, which was the most intuitive difference between the crystallization of thin films and ultrathin films. The concentration of solutions for preparing PLA/PBS blend thin films was determined as 20 mg/mL.

### 3.2. Microstructure of PLA/PBS Blend Film

**Figure 4:**
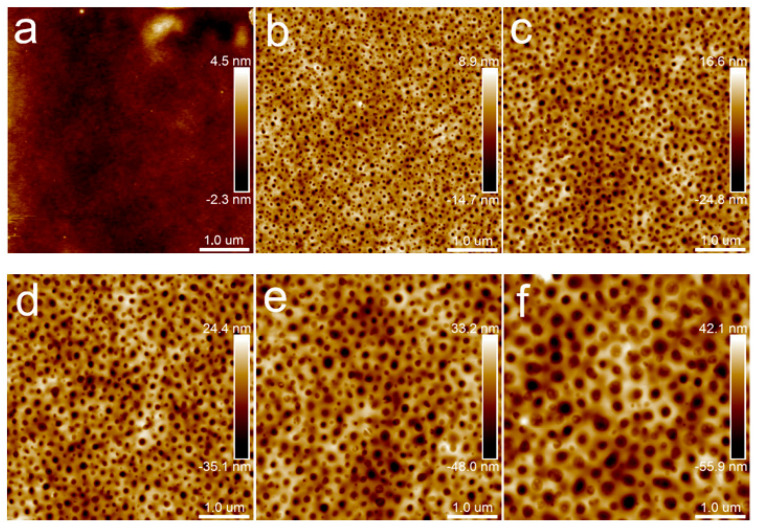
AFM topography images of PLA/PBS blend thin films without annealing: (**a**) 100/0; (**b**) 90/10; (**c**) 85/15; (**d**) 80/20; (**e**) 75/25; (**f**) 70/30. The scan size was 5 × 5 μm^2^.

Figure 4 presents a set of height images of films from neat PLA to PLA/PBS (70/30), respectively. The neat PLA film was relatively flat, as shown in Figure 4a. Obvious phase separation was observed in the composite films, due to the poor compatibility between PLA and PBS. Generally, during the spin-coating process of the blend solution, the more soluble phase collapses below the other phase as the solvent evaporates [20]. This may be attributed to the higher solubility of the PBS phase than the PLA phase in chloroform, which results in the PBS phase appearing as pores. The PBS phase had uniformly distributed pores with different diameters. With the increase in PBS content, the number of dispersed phases decreased and the size of the dispersed phases increased.

**Figure 5:**
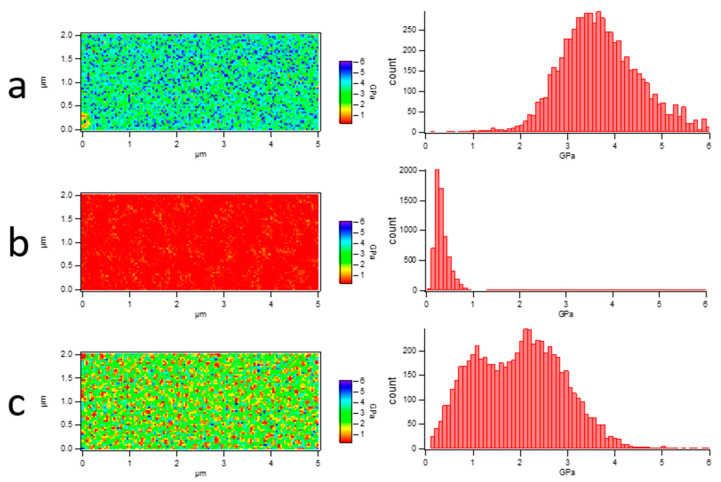
Young’s modulus map of PLA/PBS blend films and histogram of Young’s modulus distribution. (**a**) PLA; (**b**) PBS; (**c**) 80/20. The scan size was 5 × 2 μm^2^.

This is an effective method to distinguish PLA and PBS in their blend film via a modulus distribution map for their remarkedly different Young’s moduli and incompatible interfaces. The blend film microstructure was visually reflected by scanning the nanomechanical properties in AFM Force Volume mode. The nanomechanical mapping was obtained through the calculation and analysis of each force curve using Johnson–Kendall–Roberts (JKR) theory [39,40], as shown in Figure 5. It demonstrated that both nanomechanical maps of PLA and PBS neat samples showed monochromatic distribution on the whole. The Young’s modulus value of PLA is 3.72 ± 0.93 GPa, while that of PBS is 318 ± 149 MPa. The different Young’s modulus values contributed to easily distinguishing the components [41,42]. In Figure 5c, it can be seen that nanomechanical mapping is presented by the dotted area of the low modulus when the PBS component addition was at 20%, which could clearly reflect the distribution of the dispersed PBS phase in the PLA matrix. Combining the height images with nanomechanical mapping of the blend film, it was determined that the area of the dispersed phase in the height map was the PBS component. Accordingly, the morphology of the PLA/PBS blend film directly reflected the content of the PBS.

**Figure 6:**
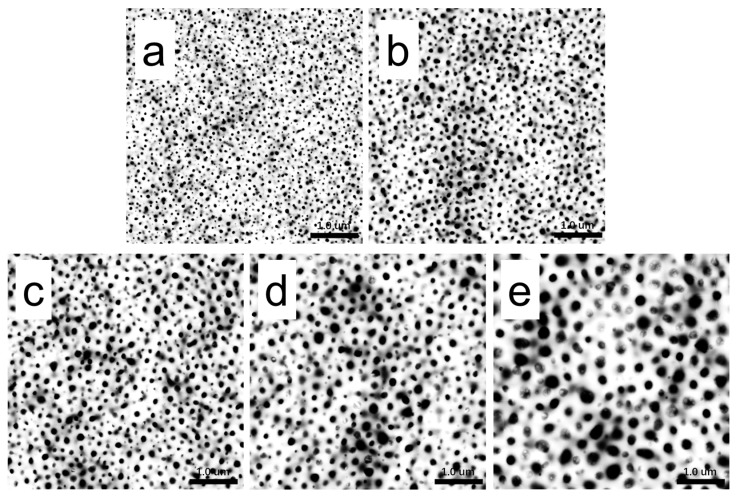
Binarization of AFM height images of PLA/PBS blend thin film at room temperature: (**a**) 90/10; (**b**) 85/15; (**c**) 80/20; (**d**) 75/25; (**e**) 70/30. The scan size was 5 × 5 μm^2^.

**Figure 7:**
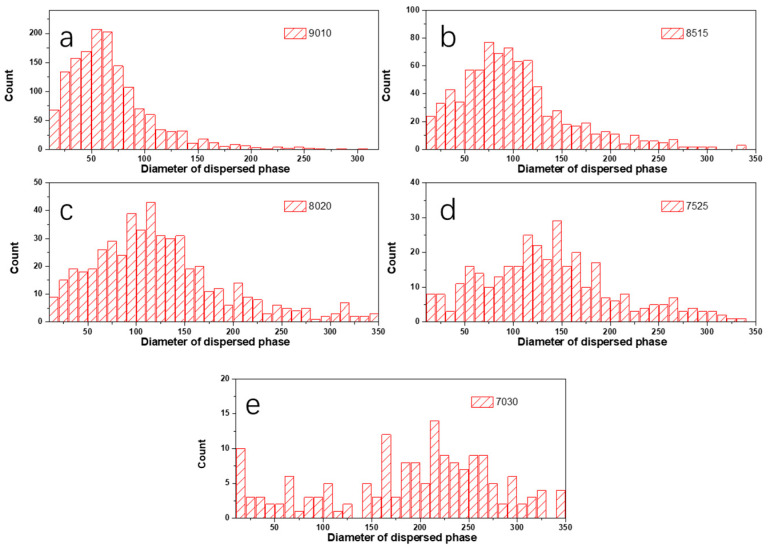
Histogram of diameter distribution of PBS phase in PLA/PBS blend films: (**a**) 90/10; (**b**) 85/15; (**c**) 80/20; (**d**) 75/25; (**e**) 70/30.

In order to analyze the dependences of the film’s microstructure on PBS content, binary processing with ImageJ was carried out on the height images of PLA/PBS blend films (Figure 4b–f), as shown in Figure 6. Statistical analysis was applied to show the relationship between the size and distribution of the dispersed phase and the content of PBS by analyzing the pictures in Figure 6 using ImageJ 1.51k [43]. As shown in Figure 7, the diameter of the dispersed phase distribution histogram in PLA/PBS blend films with different components indicated that the PBS content had a significant influence on the number and size of the dispersed phase in the film. With the PBS content increasing in the blend films, the diameter distribution range of the dispersed phase gradually widened and the median of the diameter distribution increased. Although most of the dispersed phase increased in size gradually, there was still a dispersed phase with a smaller size.

**Figure 8:**
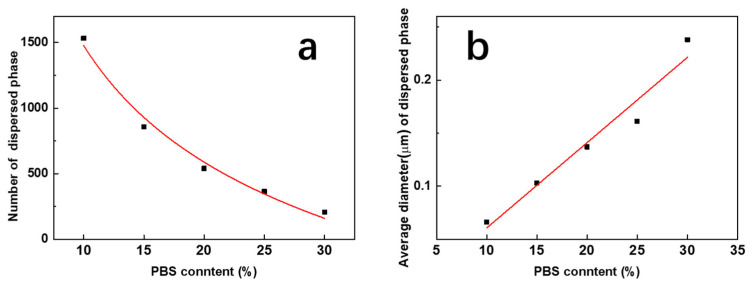
Influence of the PBS content on the total number (**a**) and average diameter (**b**) of PBS pellets induced by phase separation.

Figure 8 shows a quantitative analysis of the influence of the content of the PBS phase on the microphase structure of blend films.

It indicated that the number of dispersed phases *n* and the PBS content *x* revealed an obvious power exponential relationship, and the fitting curve function was

*n* = 10^3.6−0.04*x*^ (7)

The mean particle size of the dispersed phase *d* showed an obvious linear relationship with its content, and the linear function equation with the *R-square* of 0.9421 was fitted as

*d* = −0.02 + 0.008*x* (8)

**Figure 9:**
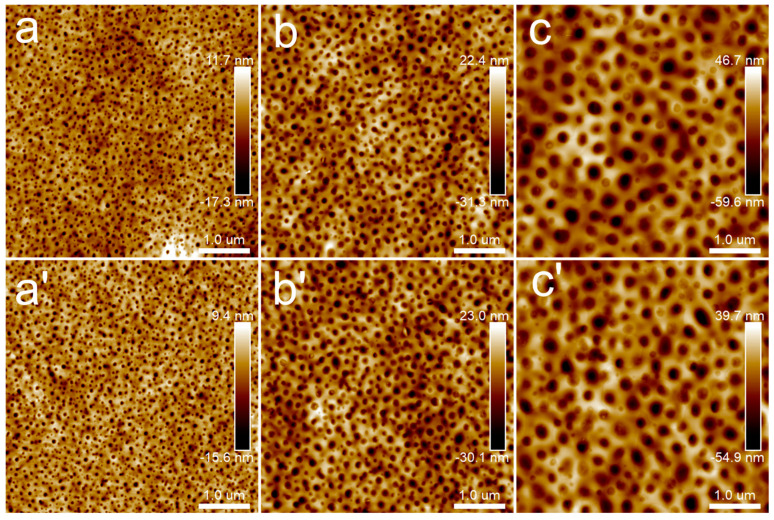
AFM topography images of PLA/PBS blend thin films with annealing at 40 °C: (**a**,**a’**) 90/10; (**b**,**b’**) 80/20; (**c**,**c’**) 70/30. The annealing time of groups (**a**–**c**) was 30 min, while that of groups (**a’**–**c’**) was 12 h. The scan size was 5 × 5 μm^2^.

Figure 9 presents topography images of different PLA/PBS blend thin films with annealing at 40 °C for 30 min and 12 h, respectively. No significant differences can be noticed on samples annealed at 40 °C for 30 min, 12 h, and without annealing (Figure 4b,d,f). This could be attributed to the factor that the lower annealing temperature (40 °C) was below the glass transition temperature (*T_g_*) (63.5 °C). The relatively low temperature restricted the PLA chain segment movement [2]. Accordingly, the morphology and structure of all the samples remained unchanged below *T_g_*.

### 3.3. Crystallization of PLA/PBS Blend Film

**Figure 10:**
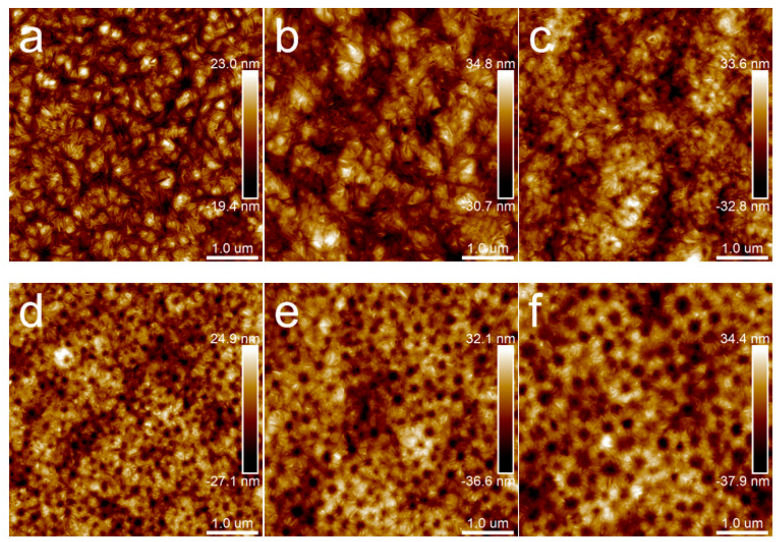
AFM topography images of PLA/PBS blend thin films with annealing at 80 °C for 30 min: (**a**) 100/0; (**b**) 90/10; (**c**) 85/15; (**d**) 80/20; (**e**) 75/25; (**f**) 70/30. The scan size was 5 × 5 μm^2^.

When the annealing temperature increased to 80 °C, the topography images of samples (Figure 10) were distinctly different from those in Figure 4 and Figure 9. When the annealing time was extended to 12 h, the results were the same as those in Figure 10. Different kinds of particular microcrystal patterns appeared, which centered on the dispersed phase and grew radioactively along the rims of the dispersed phase. Moreover, with the PBS content increasing, the dispersed phase became larger, and the feature of the microcrystals transformed from a daisy shape to a sunflower pattern.

**Figure 11:**
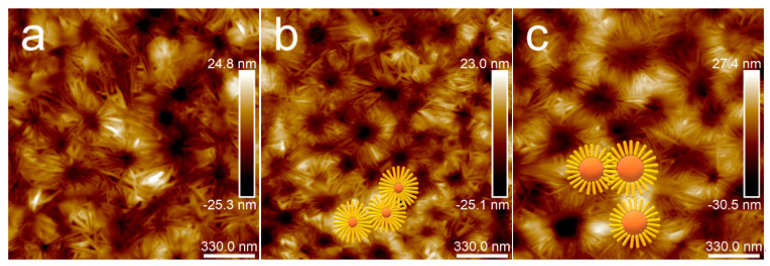
AFM topography images of PLA/PBS blend thin films with annealing at 80 °C for 30 min: (**a**) 85/15; (**b**) 80/20; (**c**) 70/30. The scan size was 1.7 × 1.7 μm^2^.

**Figure 12:**
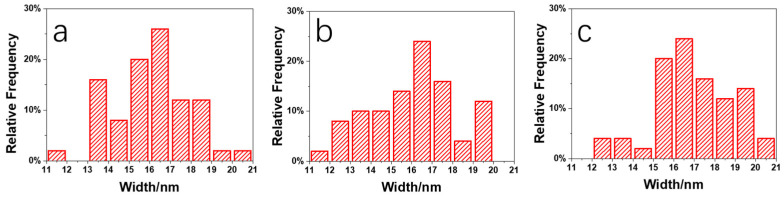
Histogram of frequency distribution of needle-shaped crystals’ width in PLA/PBS blend: (**a**) 85/15; (**b**) 80/20; (**c**) 70/30.

Figure 11 shows an enlarged height image of the PLA/PBS blend thin films annealing at 80 °C for 30 min. The frequency distribution histogram of the needle-shaped crystals’ width was obtained with the needle-shaped crystals width statistical analysis, shown in Figure 12. The needle-shaped crystal width of each sample did not change too much with the varying PBS content and the average width was approximately 16 nm. Since the PLA crystallized under the same condition, the width of the microcrystalline structure showed no difference. With the increase in PBS loading, the most obvious difference for the annealed blend films was the shape of the petals, that is, the ratio of “petals” to “stamens”.

**Figure 13:**
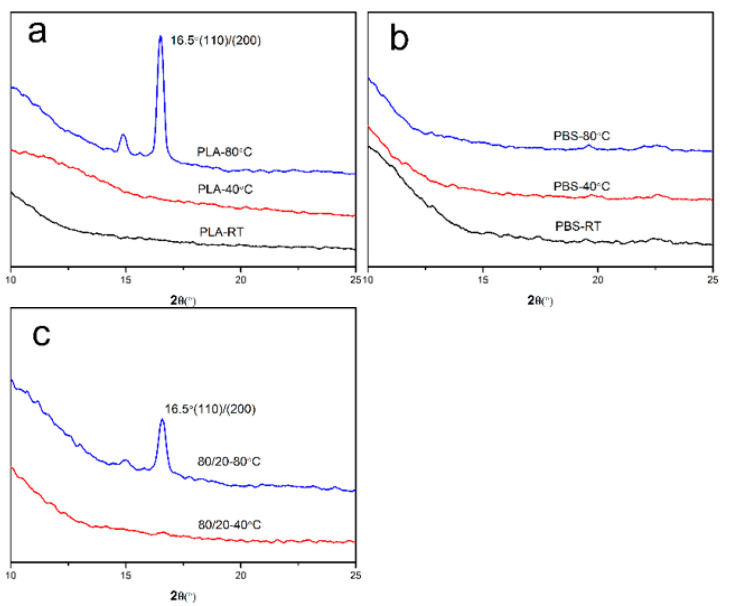
XRD patterns of PLA/PBS blend thin films with annealing at different temperatures for 30 min: (**a**) 100/10; (**b**) 0/100; (**c**) 80/20.

X-ray diffraction (XRD) was used to characterize the crystallization behavior of PLA/PBS blend films after annealing at different temperatures, as shown in Figure 13. It was found that neat PLA [44] only showed a strong diffraction peak of 16.5° after annealing at 80 °C. Nevertheless, the neat PBS films had no obvious diffraction peak. The diffraction peak of the 80/20 film appeared after annealing at 80 °C, and the position was consistent with that of neat PLA. It indicated that the PLA and PBS components in the untreated or the annealed film at 40 °C were amorphous. The petal-like pattern which formed after annealing at 80 °C could be attributed to the PLA phase crystallization [29].

**Figure 14:**
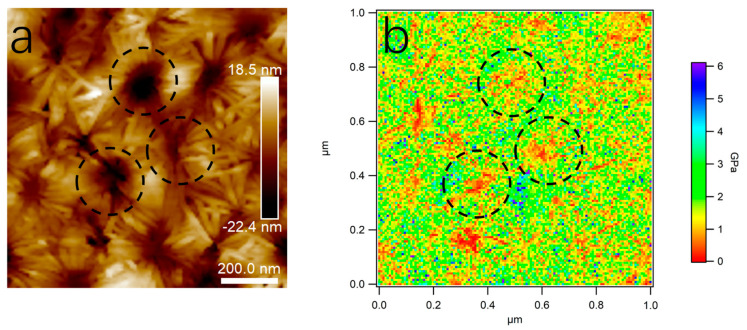
AFM height image (**a**) and Young’s modulus map (**b**) of 80/20. The scan size was 1.0 × 1.0 μm^2^.

A Young’s moduli map of the sample (80/20) was drawn to further verify the microcrystalline structure in PLA/PBS blend films. Figure 14 shows the height image of the microcrystalline structure and the corresponding Young’s moduli map. Combined with the results in Figure 5, the Young’s modulus of the PLA phase was about 3 GPa and that of the PBS phase was around 300 MPa. Thus, the microcrystalline part was the region with a high modulus, corresponding to the crystallized PLA, while the flower center part within the dotted circle was the region with a low modulus, corresponding to the amorphous PBS.

### 3.4. Mechanism of Crystallization

**Figure 15:**
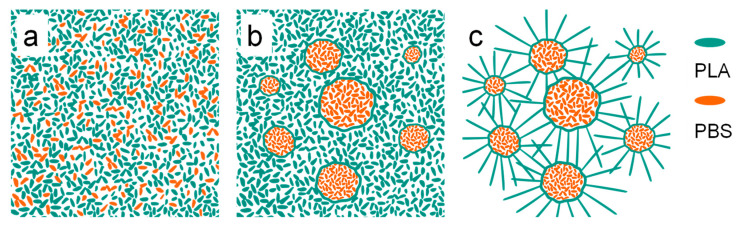
Schematic diagram of crystallization mechanism model of PLA/PBS blend films. (**a**) PLA/PBS blend solution; (**b**) separation of PLA/PBS thin films; (**c**) crystallization of PLA.

Based on these results, a possible mechanism for the petal-like pattern formation could be proposed, as shown in Figure 15. In the chloroform solution of the PLA/PBS blend, the molecular chains of both the PLA and the PBS were uniformly dispersed in the solution, as illustrated in Figure 15a. After spin casting, microphase separation happened due to the poor compatibility between the PLA and the PBS, which induced a typical sea-island structure formation. Moreover, the driving force mediated by phase separation caused the PLA molecular chain segments’ orientation along the PLA/PBS interfaces, as shown in Figure 13b. When the annealing temperature was lower than *T_g_*, the chain segment of the PLA could not move, and the morphology of PLA-based blend films was not affected. When the annealing temperature was higher than *T_g_,* the PLA molecular segment tended to crystallize in the direction perpendicular to the oriented segments under the induction of their orientation structure [22]. Eventually, it formed the petal-like pattern, where the crystallized PLA phase was the “petal” and the amorphous PBS phase was the “stamen”.

## 4. Conclusions

In summary, this work mainly reported a strategy for the petal-like patterning of PLA/PBS thin films. PLA/PBS blend films without extra annealing presented obvious phase separation and the size and number of the PBS phase had a power exponential relationship and linear relationship with PBS loading, respectively. After annealing at 80 °C for 30 min, the PLA phase formed an orderly petal-like microcrystalline structure centered on the PBS phase, and the microcrystalline morphology changed from a “daisy type” to a “sunflower type” with the increased size of the PBS phase. The pattern was controllable from a “daisy type” to a “sunflower type” with a varying ratio of the PLA “petal” and PBS “stamen”, which could be realized by changing the PBS content. The mechanism was the polymer chain orientation mediated by phase separation, which resulted in the directional crystallization of the PLA phase after annealing beyond *T_g_.* This could be used to expand the application of PLA in environmentally friendly films and devices. Since the forming process can be easily realized in an incompatible crystalline polymer blend, it might be universally applied to another polymer blend.

## Data Availability

The data presented in this study are available on request from the corresponding author.
